# Multiple exposure to environmental factors and variations in *CYP27B1* and the microRNA‐binding site of *IL‐13* are associated with breast cancer risk

**DOI:** 10.1002/cam4.2202

**Published:** 2019-04-30

**Authors:** Nannan Zhang, Yanbo Chen, Shuo Li, Huihui Yin, Liangliang Li, Ming Shan, Zhiping Long, Jingshen Tian, Jing Li, Hongyuan Yu, Kun Xie, Zhen Wu, Volontovich Daria, Fan Wang, Yashuang Zhao

**Affiliations:** ^1^ Department of Epidemiology, School of Public Health Harbin Medical University Harbin Heilongjiang Province P. R. China; ^2^ Department of Breast Surgery The Third Affiliated Hospital of Harbin Medical University Harbin Heilongjiang Province P. R. China

**Keywords:** breast cancer, *CYP27B1*, environmental factors, *IL‐13*, polymorphism

## Abstract

**Purpose:**

Several molecular epidemiology studies have evidenced an association of environmental factors and genetic polymorphisms with breast cancer (BC) risk. However, most have considered the functions of a single element rather than combined effects.

**Methods:**

This case‐control study of 693 newly‐diagnosed BC cases and 714 cancer‐free controls evaluated the effect of multiple exposures to environmental factors and polymorphisms in *CYP27B1* and *IL‐13* on BC risk. Genotypes were detected using TaqMan genotyping. Combinations and interactions were analyzed using cross‐over analysis and multivariate logistic regression. Combining exposure models were assessed using classification and regression tree and multivariate logistic regression analyses.

**Results:**

No significant independent association was observed for any polymorphism in *CYP27B1* or *IL‐13* with the risk of BC. However, significant combined effects were noted for ≥1 time/wk physical activity with rs10877012 (adjusted odds ratio [OR_adj_] = 0.21, 95% confidence interval [CI] = 0.11‐0.39) and rs4646536 (OR_adj_ = 0.21, 95% CI = 0.11‐0.39) in *CYP27B1*. Furthermore, taking garlic ≥4 times/wk, ≥1 time/wk physical activity, and a psychological index score ≥33 all displayed significant combined effects with three *IL‐13* polymorphisms. These relationships remained significant after Bonferroni correction for multiple comparisons. Combining exposure models indicated that compared with consuming garlic ≥4 times/wk, five models (model 5, OR_adj_ = 2.94, 95% CI = 1.07‐8.06; model 6, OR_adj_ = 10.26, 95% CI = 5.81‐18.10; model 7, OR_adj_ = 5.05, 95% CI = 2.78‐9.17; model 8, OR_adj_ = 3.95, 95% CI = 2.79‐5.58; and model 9, OR_adj_ = 7.97, 95% CI = 5.26‐12.07) showed a significant increased risk.

**Conclusions:**

Our findings suggest that personalized adjustments to diet and behavioral patterns may aid BC prevention in variant carriers of *CYP27B1* and *IL‐13*.

## 1 INTRODUCTION

Breast cancer (BC) is a major malignant disease that threatens women's health. The American Cancer Society estimated that the number of new cases of female BC would reach 2.1 million worldwide in 2018, and that 626 679 deaths from BC would occur.^1^ Breast cancer was also reported to be the leading cause of cancer death among women in developing countries.^1^ Approximately 268 600 new BC patients and 69 500 BC deaths in female occurred in China in 2015.^2^

Breast cancer is known to be predominantly caused by environmental factors, such as diet (the consumption of meat, fruit, and vegetables), excessive drinking and smoking, and a lack of physical exercise,^3^, ^4^ especially in sporadic patients. Moreover, some aromatic hydrocarbons in tobacco and increased polycyclic aromatic hydrocarbons in barbecued meat may act directly as carcinogens or indirectly modify the individual risk of BC‐associated genetic polymorphisms.^5^, ^6^ Most previous epidemiological studies have focused on the modified risk of a single environmental factor, so the challenge is to recognize and interpret combined exposure effects and translate from concept to utility to better delineate the causes and provide personalized prevention for BC. Recently, Wild et al^7^ defined the exposome and drew attention to the need for more complete environmental exposure assessment in epidemiological studies. They indicated that information should be gathered to investigate not only the link between one type of exposure and a disease, but also to provide the measurement for combinations of multiple exposures and yield multiple exposure models associated with disease.

Vitamin D, present in the blood as 25‐hydroxyvitamin D_3 _(25(OH)D_3_), was shown to be associated with a reduced risk of female BC.^8^ *CYP27B1* encodes a member of the cytochrome P450 family, which catalyzes the conversion of 25(OH)D_3_ to 1‐alpha,25‐dihydroxyvitamin D3 (1α,25(OH)_2_D3); this then binds to the vitamin D receptor to promote bone resorption and regulate cell proliferation and differentiation.^9^, ^10^, ^11^ Interleukin 13 (encoded by *IL‐13*) is a critical immune regulatory cytokine secreted predominantly by activated T‐helper 2 lymphocytes.^12^ *IL‐13* plays an important role in lymphocyte proliferation and activation and can induce apoptosis.^13^ Additionally, microRNAs (miRNAs), endogenous noncoding nucleotide sequences, function in gene regulation by binding to targeted mRNAs in 3′‐untranslated regions (UTRs) to alter mRNA levels and protein expression.^14^ Single nucleotide polymorphisms (SNPs) located within miRNA‐binding sites could therefore influence cancer risk and recurrence.^15^, ^16^, ^17^ Several studies have focused on the association between polymorphisms in *CYP27B1* and *IL‐13* with cancer risk,^18^, ^19^ but little is known about polymorphisms in these genes and miRNA‐binding sites with respect to BC risk.

Therefore, the present study investigated the association of polymorphisms in *CYP27B1* and the miRNA‐binding site of *IL‐13* with the risk of BC, and assessed the combination and interaction between these and possible environmental exposure on BC. The effects of multiple exposures to environmental factors and polymorphisms on BC were also examined, together with a correlation between these polymorphisms and clinical characteristics.

## 2 MATERIALS AND METHODS

### 2.1 Study subjects

This case‐control study included 693 newly‐diagnosed BC cases and 714 cancer‐free controls. Cases were pathologically diagnosed patients collected from the Third Affiliated Hospital of Harbin Medical University during October 2011 to May 2014. Any patient who had undergone radiotherapy or chemotherapy was excluded. Control subjects were selected from the Department of Orthopaedics and Ophthalmology at the First and Second Affiliated Hospitals of Harbin Medical University, HongQi community of Harbin and XiangFang District Centers for Disease Control and Prevention in the same period (187 community‐based and 527 hospital‐based). Any individual with a history of BC or malignancies and benign neoplasms was excluded. We also excluded postpartum or lactating women and pregnant women from both cases and controls. Approximately 5 mL of fasting peripheral venous blood was obtained from all participants, before surgery for patients or at enrollment for controls.

All procedures performed in studies involving human participants were in accordance with the ethical standards of the Human Research and Ethics Committee of Harbin Medical University and with the 1964 Helsinki declaration and its later amendments or comparable ethical standards. Informed consent was obtained from all individual participants included in the study.

### 2.2 Data collection

A structured questionnaire was designed to collect information from each subject using a face‐to‐face approach by well‐trained interviewers. The questionnaire included questions on demographic characteristics (age, height, weight, marital status, education, occupation, and family history of cancer), lifestyle (smoking, drinking, and physical activity), frequency of consumption of food items (cereals, garlic, poultry, pork, fish, milk, soybean, overnight food, eggs, canned fruit, canned meat, coffee, carbonated drinks, and juice), and psychological status. Lifestyle and dietary details were recalled for the last year before cancer diagnosis. We also obtained clinicopathological information about each patient including tumor location, maximum diameter, molecular classification, tumor stage (T stage), lymph node stage (N stage), metastasis stage (M stage), and levels of estrogen receptor (ER), progesterone receptor (PR), human epidermal growth factor receptor 2 (HER2), and carbohydrate antigen‐153 (CA‐153).

### 2.3 SNP selection and genotyping

We used the dbSMR database to conduct an extensive search on SNPs located in miRNA‐binding sites within the 3′‐UTRs of *CYP27B1* and *IL‐13* (http://miracle.igib.res.in/polyreg/). The RNAhybrid tool (http://bibiserv.techfak.uni-bielefeld.de/rnahybrid/submission.html) was applied to assess the Gibbs free energy of binding (DG, expressed in kJ/mol) both for wild‐type and variant alleles. DDG was calculated as the difference of DG between the two alleles (wild‐type allele DG vs variant allele DG). The absolute values of DDG (|DDG|) were used to avoid positive and negative DDG values negating each other. Finally, the |DDG tot| values (sums of all |DDG|s for each SNP) were computed as parameters to predict the biological effects of the variations. The top SNPs in *IL‐13* were selected for genotyping. No polymorphisms in miRNA target sites in *CYP27B1* were found, but two common polymorphisms (rs10877012 and rs4646536) with a minor allele frequency of more than 5% were selected based on previous studies.^20^ Detailed information on these five genetic polymorphisms is provided in Table S1.

We extracted genomic DNA from peripheral blood leukocyte cell pellets using the QIAamp DNA Blood Mini Kit (QIAGEN, Valencia, CA, USA) according to the manufacturer's instructions. DNA samples were analyzed using the fluorogenic 5′‐nuclease assay (TaqMan SNP Genotyping Assay; Applied Biosystems, Foster City, CA) using a Lightcycler^®^ 480|| (Roche, Applied Biosystems) platform. Probe assay IDs were as follows: rs10877012: C_25623453_10; rs4646536: AHCTA6I; rs847: C_8932046_10; rs848: C_8932051_20; and rs1295685: C_8932052_10. According to the protocol, the 25 μL reaction mix consisted of at least 10 ng of DNA, 12.5 μL of Universal PCR master mix, and 0.625 μL of probe/primer mix. Polymerase chain reaction (PCR) conditions were 95°C for 10 minutes, followed by 40 cycles of 92°C for 15 seconds and 60°C for 1 minute.

### 2.4 Statistical analysis

The Hardy‐Weinberg equilibrium (HWE) was adopted to calculate genotype distributions in the controls. Chi‐square test for categorical variables and two‐sample *t* test for continuous variables were selected, respectively. The Akaike information criterion (AIC) statistic was used to evaluate the goodness of model fitting. The model with the lowest AIC value was considered the best one and was analyzed in interaction and combined analyses. We used the Bonferroni‐corrected *P*‐value for multiple corrections. Haplotypes were evaluated by SHEsis software (http://analysis.bio-x.cn/myAnalysis.php). Crude and adjusted odds ratios (ORs) and 95% confidence intervals (95% CIs) were calculated using univariate and multivariate logistic regression analyses. The exposure combination models were generated using classification and regression tree (CART) with pruning using the statistical software package R Studio. Logistic regression analyses were adopted to assess the relationships of these models and BC risk. Other statistical analyses were carried out using sas software version 9.2 (SAS Institute, Cary, NC). All reported *P* values were two‐sided, and *P* < 0.05 was considered significant.

## 3 RESULTS

### 3.1 Demographic characteristics

Table 1 shows the demographic characteristics of 693 cases and 714 controls. The mean (±SD) age of BC patients and controls was 52.20 ± 9.61 and 53.20 ± 10.65 years, respectively (*P* = 0.038). We also observed significant differences between cases and controls regarding education level (*P* < 0.001), marriage status (*P* = 0.034), and family history of cancer (*P* < 0.001). Therefore, we chose these four characteristics as adjusted factors for multivariate analyses. No significant differences were found with regard to BMI (body mass index).

**Table 1:** Characteristics of breast cancer cases and controls

Variable^a^	Case no. (%)	Control no. (%)	*P*‐value
	693	714	
Age (y)			**0.038**
<50	298 (43.06)	262 (36.80)	
50‐59	245 (35.40)	267 (37.50)	
60‐69	116 (16.76)	130 (18.26)	
≥70	33 (4.77)	53 (7.44)	
Mean ± SD	52.20 ± 9.61	53.20 ± 10.65	
BMI^b^			0.226
≤18.5	26 (3.78)	40 (5.76)	
18.5‐23	262 (38.14)	260 (37.46)	
>23	399 (58.08)	394 (56.77)	
Mean ± SD	23.99 ± 4.67	16.37 ± 11.46	
Educational level			**0.000**
Primary school	185 (26.77)	337 (48.07)	
Middle school	218 (31.55)	189 (26.96)	
High school	183 (26.48)	134 (19.12)	
College	105 (15.20)	41 (5.85)	
Marriage status^c^			**0.034**
Single	3 (0.44)	12 (1.71)	
Married	680 (99.56)	689 (98.29)	
Family history of cancer			**0.000**
Yes	12 (1.89)	58 (8.59)	
No	623 (98.11)	617 (91.41)	

Bold values indicate statistical significance after Bonferroni correction.

a Missing data: age: 1 case, 2 controls; marriage status: 10 cases, 13 controls; cultural level: 2 cases, 13 controls; BMI: 6 cases, 20 controls; family history of cancer: 58 cases, 39 controls.

b BMI, body mass index (weight/height^2^).

c Marriage status: single is the person who has not married. Married is the person who has married or cohabitated, including widowed (not remarried), separated (due to discord or long‐distance), and divorced (not remarried).

### 3.2 Polymorphisms in *CYP27B1* and *IL‐13* and the risk of BC

Table S1 shows the location of five SNPs in *CYP27B1* and *IL‐13*. Two (rs10877012, rs4646536) of these were intronic and near the 5′ region of *CYP27B1*, and three (rs847, rs848, and rs1295685) were located in miRNA‐binding sites of *IL‐13*. Table S1 also lists the sum of |ΔΔG| values for each SNP.

Table S2 shows that the genotype distributions of all five polymorphisms in the controls were in accordance with HWE (*P* > 0.05). The genotype distribution of the five polymorphisms and their adjusted ORs and 95% CIs for the risk of BC are shown in Table 2. For *CYP27B1* rs10877012, the frequencies of GG, GT, and TT were 40.50%, 44.15%, and 15.35% in controls, and 42.96%, 44.72%, and 12.32% in cases, respectively. The frequencies of genotypes in *CYP27B1* rs4646536 were 40.47% for CC, 44.10% for CT, and 15.43% for TT among controls, and 43.34% for CC, 44.36% for CT, and 12.30% for TT among cases. We observed no significant associations with BC risk for these two SNPs. Similarly, no meaningful associations were detected between the miRNA‐binding site SNPs rs847, rs848, and rs1295685 of *IL‐13* and the risk of BC. In view of the AIC values, the recessive model for rs10877012 and rs4646536, and the dominant model for rs847, rs848, and rs1295685 were chosen in interaction analysis and multivariate regression analyses.

**Table 2:** Associations between the polymorphisms in *CYP27B1, IL‐13* and the risk of breast cancer

Genotype^a^	Cases no. (%)	Controls no. (%)	OR_adj_ (95% CI)	*P‐*value	P* value	AIC
*CYP27B1* G>T (rs10877012)						
GG	293 (42.96)	277 (40.50)	1.00			
GT	305 (44.72)	302 (44.15)	0.98 (0.76‐1.26)	0.880	1.000	
TT	84 (12.32)	105 (15.35)	0.80 (0.55‐1.15)	0.232	1.000	
Dominant model			0.93 (0.74‐1.18)	0.568	1.000	1635.02
Recessive model			0.81 (0.58‐1.14)	0.229	1.000	1633.83
Addictive model			0.92 (0.78‐1.09)	0.314	1.000	1635.81
*CYP27B1 C>T* (rs4646536)						
CC	296 (43.34)	278 (40.47)	1.00			
CT	303 (44.36)	303 (44.10)	0.97 (0.75‐1.24)	0.797	1.000	
TT	84 (12.30)	106 (15.43)	0.79 (0.55‐1.13)	0.197	1.000	
Dominant model			0.92 (0.73‐1.16)	0.486	1.000	1641.88
Recessive model			0.81 (0.58‐1.13)	0.212	1.000	1640.69
Addictive model			0.91 (0.77‐1.07)	0.263	1.000	1642.62
*IL‐13 A>G* (rs847)						
AA	317 (47.81)	328 (47.61)	1.00			
AG	290 (43.74)	311 (45.14)	1.12 (0.87‐1.42)	0.379	1.000	
GG	56 (8.45)	50 (7.26)	1.29 (0.81‐2.04)	0.280	1.000	
Dominant model			1.14 (0.90‐1.43)	0.288	1.000	1619.64
Recessive model			1.22 (0.78‐1.90)	0.381	1.000	1620.05
Addictive model			1.12 (0.93‐1.35)	0.224	1.000	1621.27
*IL‐13 G>T* (rs848)						
GG	323 (47.96)	329 (47.85)	1.00			
GT	294 (43.29)	297 (43.56)	1.14 (0.89‐1.45)	0.308	1.000	
TT	58 (8.75)	60 (8.59)	1.11 (0.72‐1.71)	0.640	1.000	
Dominant model			1.13 (0.90‐1.42)	0.305	1.000	1637.03
Recessive model			1.05 (0.69‐1.59)	0.822	1.000	1638.06
Addictive model			1.09 (0.91‐1.30)	0.369	1.000	1639.02
*IL‐13 C>T* (rs1295685)						
CC	320 (47.44)	324 (47.34)	1.00			
CT	292 (43.63)	298 (43.20)	1.10 (0.86‐1.40)	0.457	1.000	
TT	64 (8.93)	61 (9.47)	1.25 (0.82‐1.90)	0.309	1.000	
Dominant model			1.12 (0.88‐1.41)	0.357	1.000	1632.83
Recessive model			1.19 (0.79‐1.78)	0.413	1.000	1633.05
Addictive model			1.10 (0.92‐1.32)	0.283	1.000	1634.49

Abbreviations: CI, confidence interval; OR, odds ratio; OR_adj,_, adjusted by age, educational level, marriage status and family history of cancer; P*, *P* values after Bonferroni correction.

a Missing values: rs10877012, 41; rs4646536, 37; rs847, 55; rs848, 46; rs1295685, 48.

### 3.3 Haplotype analyses of *CYP27B1* and *IL‐13* and BC risk

We calculated haplotypes and their frequencies among cases and controls for the five SNPs (Table S3). Haplotypes with frequencies <3% were excluded. G‐C of *CYP27B1* accounted for the largest proportion in cases (65.30%) and controls (62.40%), while A‐G‐C of *IL‐13* made up a relatively high proportion (67.00% in cases and 66.80% in controls). However, no noteworthy associations of haplotypes and BC risk were detected.

### 3.4 Combined and interactive effects of polymorphisms and environmental factors on the risk of BC

We analyzed the associations between all environmental factors in our questionnaire and the risk of BC. Table S4 shows the results from univariate and multivariate logistic regression for environmental factors. Among those factors, taking >200 g/wk cereal (OR_adj_ = 0.33, 95% CI = 0.20‐0.56), ≥4 times/wk garlic (OR_adj_ = 0.23, 95% CI = 0.15‐0.36), and ≥1 time/wk physical activity (OR_adj_ = 0.43, 95% CI = 0.32‐0.60) reduced BC risk. Higher consumption of poultry, milk, and soybean (all *P* < 0.05) was also associated with a decreased risk of BC. By contrast, higher consumption of pork, >3 times/wk overnight food (OR_adj_ = 1.64, 95% CI = 1.08‐2.51), and a higher psychological index score (≥33, OR_adj_ = 2.39, 95% CI = 1.48‐3.86) were significantly associated with an increased risk of BC.

Only those environmental factors identified as significant by multivariate analyses were examined for their combined and interactive effects with polymorphisms in relation to BC (Tables 3 and 4). In a recessive model of *CYP27B1* rs10877012, we observed valuable combined effects for the TT genotype and physical activity (OR_adj_ = 0.21, 95% CI = 0.11‐0.39). This effect was also found to have statistical significance for the TT genotype in *CYP27B1* rs4646536 (recessive model) and ≥1 time/wk physical activity (OR_adj_ = 0.21, 95% CI = 0.11‐0.39). This indicated that performing physical activity ≥1 time/wk could have more beneficial effects on reducing BC risk in carriers of homozygous variants in these two *CYP27B1* polymorphisms. No significant interactive effect was observed between *CYP27B1* polymorphisms and environmental factors on BC risk.

**Table 3:** Combined and interactive effects between polymorphisms in *CYP27B1* and environmental factors in breast cancer

Environmental factors	rs10877012 (Recessive model)		Interaction		rs4646536 (Recessive model)		Interaction	
	GG + GT	TT	OR_i_ (95% CI)	*P*	CC + CT	TT	OR_i_ (95% CI)	*P*
	OR_adj_ (95% CI)				OR_adj_ (95% CI)			
Cereal (g/wk)			2.41 (0.58‐9.99)	0.225			2.94 (0.68‐14.77)	0.150
<200	1.00	0.67 (0.45‐0.97)			1.00	0.65 (0.45‐0.95)		
≥200	0.39 (0.25‐0.62)	0.67 (0.18‐2.52)			0.38 (0.24‐0.60)	0.77 (0.20‐3.02)		
Garlic (time/wk)			2.62 (1.12‐6.12)	0.027			2.42 (1.04‐5.63)	0.040
<4	1.00	0.67 (0.46‐0.99)			1.00	0.68 (0.46‐1.00)		
≥4	0.23 (0.16‐0.32)	0.39 (0.19‐0.80)			0.23 (0.16‐0.32)	0.37 (0.18‐0.75)		
Poultry			2.45 (1.23‐4.90)	0.011			2.31 (1.16‐4.62)	0.018
No	1.00	0.51 (0.31‐0.83)			1.00	0.53 (0.32‐0.86)		
Yes	0.44 (0.34‐0.57)	0.53 (0.32‐0.87)			0.45 (0.34‐0.58)	0.53 (0.32‐0.86)		
Pork (g/wk)			1.02 (0.51‐2.06)	0.947			1.02 (0.51‐2.05)	0.962
<250	1.00	0.79 (0.50‐1.26)			1.00	0.80 (0.51‐1.27)		
≥250	0.97 (0.75‐1.27)	0.76 (0.45‐1.29)			0.96 (0.74‐1.25)	0.75 (0.45‐1.27)		
Milk (times/wk)			0.74 (0.35‐1.54)	0.417			0.74 (0.35‐1.54)	0.423
<1	1.00	0.82 (0.53‐1.27)			1.00	0.80 (0.52‐1.24)		
≥1	0.52 (0.40‐0.67)	0.31 (0.17‐0.56)			0.53 (0.41‐0.68)	0.31 (0.17‐0.57)		
Soybean (times/wk)			1.89 (0.94‐3.79)	0.075			1.93 (0.96‐3.87)	0.064
≤1	1.00	0.56 (0.33‐0.94)			1.00	0.55 (0.33‐0.93)		
>1	0.67 (0.52‐0.87)	0.70 (0.43‐1.13)			0.68 (0.53‐0.89)	0.71 (0.44‐1.15)		
Overnight food (times/wk)^a^			0.84 (0.41‐1.70)	0.621			0.83 (0.41‐1.69)	0.606
≤3	1.00	0.83 (0.54‐1.28)			1.00	0.83 (0.54‐1.27)		
>3	1.81 (1.39‐2.37)	1.27 (0.73‐2.22)			1.82 (1.39‐2.38)	1.25 (0.72‐2.20)		
Physical activity (times/wk)			0.54 (0.25‐1.16)	0.113			0.54 (0.25‐1.15)	0.112
<1	1.00	1.02 (0.65‐1.60)			1.00	1.00 (0.64‐1.58)		
≥1	0.42 (0.31‐0.55)	**0.21 (0.11‐0.39)**			0.42 (0.32‐0.56)	**0.21 (0.11‐0.39)**		
Psychological index^b^			2.68 (0.96‐7.52)	0.061			2.73 (0.98‐7.67)	0.056
<33	1.00	0.68 (0.46‐1.01)			1.00	0.67 (0.46‐0.99)		
≥33	2.16 (1.56‐2.98)	4.17 (1.65‐10.54)			2.15 (1.56‐2.97)	4.19 (1.66‐10.59)		

Note:

We analyzed the combined effects for 10 environmental factors and five polymorphisms of *CYP27B1* and *IL‐13*, the *P* value after Bonferroni correction is 0.05/45 = 0.0001. We analyzed the interactive effects using logistic regression, the significant *P* value of interactive effect is 0.05.

Bold values indicate significance after Bonferroni correction.

Abbreviations: OR_adj_, adjusted for age, educational level, marriage status and family history of cancer; OR_i_, OR_interaction_, interactive effects.

a Overnight food, the vegetables, eggs, meat that have been cooked and left overnight.

b Psychological index was evaluated using psychosocial stress survey for groups (PSSG).

**Table 4:** Combined and interactive effects between polymorphisms in *IL‐13* and environmental factors in breast cancer

Environmental factors	rs847 (Dominate model)		Interaction		rs848 (Dominant model)		Interaction		rs1295685 (Dominant model)		Interaction	
	AA	AG + GG	OR_i_ (95% CI)	*P*	GG	GT + TT	OR_i_ (95% CI)	*P*	CC	CT + TT	OR_i_ (95% CI)	*P*
	OR_adj_ (95% CI)				OR_adj_ (95% CI)				OR_adj_ (95% CI)			
Cereal (g/wk)			0.81 (0.35‐1.89)	0.622			0.78 (0.34‐1.80)	0.558			1.75 (0.33‐1.74)	0.505
<200	1.00	1.14 (0.88‐1.48)			1.00	1.16 (0.89‐1.50)			1.00	1.16 (0.89‐1.50)		
≥200	0.46 (0.25‐0.85)	0.44 (0.24‐0.81)			0.48 (0.26‐0.88)	0.45 (0.25‐0.81)			0.48 (0.27‐0.88)	0.43 (0.24‐0.78)		
Garlic (times/wk)			0.72 (0.39‐1.35)	0.307			0.72 (0.38‐1.33)	0.289			0.57 (0.30‐1.06)	0.074
<4	1.00	1.23 (0.94‐1.61)			1.00	1.23 (0.94‐1.60)			1.00	1.29 (0.99‐1.69)		
≥4	0.30 (0.20‐0.48)	**0.27 (0.17‐0.42)**			0.31 (0.20‐0.49)	**0.28 (0.18‐0.43)**			0.34 (0.22‐0.53)	**0.25 (0.16‐0.39)**		
Poultry			0.90 (0.56‐1.46)	0.673			0.88 (0.54‐1.43)	0.605			0.88 (0.54‐1.42)	0.589
No	1.00	1.19 (0.84‐1.67)			1.00	1.19 (0.85‐1.68)			1.00	1.25 (0.89‐1.76)		
Yes	0.53 (0.37‐0.75)	0.59 (0.42‐0.83)			0.51 (0.36‐0.73)	0.56 (0.40‐0.79)			0.53 (0.37‐0.74)	0.60 (0.43‐0.85)		
Pork (g/wk)			1.06 (0.65‐1.72)	0.827			1.05 (0.65‐1.70)	0.850			0.98 (0.60‐1.59)	0.928
<250	1.00	1.11 (0.82‐1.52)			1.00	1.11 (0.82‐1.51)			1.00	1.18 (0.87‐1.61)		
≥250	0.95 (0.67‐1.35)	1.17 (0.82‐1.66)			0.94 (0.66‐1.33)	1.13 (0.79‐1.60)			0.98 (0.69‐1.39)	1.17 (0.83‐1.66)		
Milk (times/wk)			1.09 (0.68‐1.77)	0.715			1.06 (0.66‐1.71)	0.803			1.04 (0.64‐1.67)	0.881
<1	1.00	1.05 (0.76‐1.44)			1.00	1.06 (0.77‐1.45)			1.00	1.06 (0.78‐1.46)		
≥1	0.49 (0.35‐0.70)	0.58 (0.41‐0.81)			0.49 (0.35‐0.69)	0.57 (0.40‐0.79)			0.51 (0.36‐0.71)	0.57 (0.41‐0.80)		
Soybean (times/wk)			0.90 (0.56‐1.45)	0.654			0.92 (0.57‐1.48)	0.726			1.42 (0.88‐2.28)	0.151
≤1	1.00	1.25 (0.87‐1.80)			1.00	1.23 (0.86‐1.77)			1.00	0.94 (0.65‐1.35)		
>1	0.78 (0.55‐1.10)	0.88 (0.63‐1.23)			0.77 (0.55‐1.08)	0.87 (0.62‐1.21)			0.62 (0.44‐0.87)	0.82 (0.59‐1.16)		
Overnight food (times/wk)^a^			0.99 (0.61‐1.62)	0.971			1.00 (0.62‐1.64)	0.986			1.03 (0.63‐1.68)	0.898
≤3	1.00	1.17 (0.87‐1.58)			1.00	1.17 (0.87‐1.57)			1.00	1.13 (0.84‐1.52)		
>3	1.88 (1.32‐2.69)	2.09 (1.47‐2.98)			1.82 (1.28‐2.59)	2.04 (1.43‐2.91)			1.77 (1.24‐2.53)	1.99 (1.40‐2.83)		
Physical activity (times/wk)			0.72 (0.44‐1.17)	0.185			0.69 (0.42‐1.13)	0.136			0.69 (0.42‐1.13)	0.143
<1	1.00	1.27 (0.93‐1.74)			1.00	1.26 (0.92‐1.72)			1.00	1.25 (0.91‐1.72)		
≥1	0.44 (0.30‐0.64)	**0.44 (0.31‐0.63）**			0.44 (0.30‐0.64)	**0.42 (0.29‐0.60)**			0.43 (0.30‐0.63)	**0.42 (0.29‐0.60)**		
Psychological index^b^			0.76 (0.42‐1.41)	0.390			0.76 (0.41‐1.39)	0.369			0.74 (0.40‐1.36)	0.325
<33	1.00	1.19 (0.92‐1.56)			1.00	1.19 (0.91‐1.55)			1.00	1.19 (0.92‐1.55)		
≥33	2.69 (1.70‐4.25)	**2.48 (1.64‐3.74)**			2.69 (1.70‐4.24)	**2.43 (1.61‐3.66)**			2.77 (1.76‐4.36)	**2.44 (1.61‐3.71)**		

Note:

We analyzed the combined effects for 10 environmental factors and five polymorphisms of *CYP27B1* and *IL‐13*, the *P* value after Bonferroni correction is 0.05/45 = 0.0001. We analyzed the interactive effects using logistic regression, the significant *P* value of interactive effect is 0.05.

Bold values indicate significance after Bonferroni correction.

Abbreviations: OR_adj_, adjusted for age, educational level, marriage status and family history of cancer; OR_i_, OR_interaction_, interactive effects

a Overnight food, the vegetables, eggs, meat that have been cooked and left overnight.

b Psychological index was evaluated using psychosocial stress survey for groups (PSSG).

Using a dominant model for *IL‐13* polymorphisms rs847, rs848, and rs1295685, taking garlic ≥4 times/wk (OR_adj_ = 0.27, 95% CI = 0.17‐0.42), physical activity ≥ 1 time/wk (OR_adj_ = 0.44, 95% CI = 0.31‐0.63), and a psychological index score ≥33 (OR_adj_ = 2.48, 95% CI = 1.64‐3.74) all displayed significant combined effects. This indicated that garlic intake and physical activity played protective effects against BC in the carriers of variants in these three *IL‐13* SNPs. Additionally, compared with individuals with a psychological index score <33, those with a score ≥33 had more than twofold risk of BC. However, variants in the three *IL‐13* SNPs were associated with a reduced increase in BC risk in individuals with a psychological index score ≥33. No significant interactive effect was found between *IL‐13* polymorphisms and environmental factors regarding BC risk.

### 3.5 Multiple exposure model analyses in BC risk

**Figure 1:**
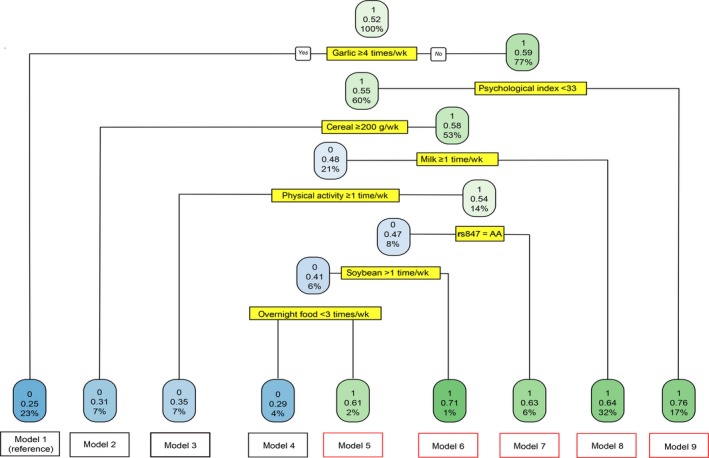
Classification and regression tree analysis for the risk of breast cancer in different exposure models. Several variables (including garlic; psychological index; cereal; milk; physical activity; rs847; soybean; overnight food) were filtered as nodes due to their significant associations with breast cancer risk. Complexity parameter of the tree is 0.01

The results of CART suggested that eight factors, including psychological index, physical activity, rs847, and the consumption of garlic, cereal, milk, soybean, and overnight food, were significantly associated with a modified risk of BC. Thus, we calculated the adjusted ORs and 95% CIs for the nine combining exposure models. Including all these factors into multivariate logistic regression analysis, significant results were observed for five types of combining exposure models. As shown in Figure 1 and Table 5, when considering the population group of garlic ≥4 times/wk as the reference, significant increased risks were detected in model 5 (OR_adj_ = 2.94, 95% CI = 1.07‐8.06), model 6 (OR_adj_ = 10.26, 95% CI = 5.81‐18.10), model 7 (OR_adj_ = 5.05, 95% CI = 2.78‐9.17), model 8 (OR_adj_ = 3.95, 95% CI = 2.79‐5.58), and model 9 (OR_adj_ = 7.97, 95% CI = 5.26‐12.07). The multiple exposures in model 6 showed the highest increased risk of BC.

**Table 5:** Multiple exposure models analyses on the risk of breast cancer

Models		OR_adj_ (95% CI)	*P‐*value
1	Garlic ≥ 4 times/wk	1.00	
2	Garlic < 4 times/wk and psychological index < 33 and cereal ≥ 200 g/wk	1.07 (0.63‐1.83)	0.797
3	Garlic < 4 times/wk and psychological index < 33 and cereal < 200 g/wk and milk ≥ 1 time/wk and physical activity ≥ 1 time/wk	1.41 (0.81‐2.44)	0.219
4	Garlic < 4 times/wk and psychological index < 33 and cereal < 200 g/wk and milk ≥ 1 time/wk and physical activity < 1 time/wk and rs847 = AA and soybean > 1 time/wk and overnight food < 3 times/wk	1.18 (0.53‐2.61)	0.685
5	Garlic < 4 times/wk and psychological index < 33 and cereal < 200 g/wk and milk ≥ 1 time/wk and physical activity < 1 time/wk and rs847 = AA and soybean > 1 time/wk and overnight food ≥ 3 times/wk	2.94 (1.07‐8.06)	**0.036**
6	Garlic < 4 times/wk and psychological index < 33 and cereal < 200 g/wk and milk ≥ 1 time/wk and physical activity < 1 time/wk and rs847 = AA and soybean ≤ 1 time/wk	10.26 (5.81‐18.10)	**0.000**
7	Garlic < 4 times/wk and psychological index < 33 and cereal < 200 g/wk and milk ≥ 1 time/wk and physical activity < 1 time/wk and rs847 = AG or GG	5.05 (2.78‐9.17)	**0.000**
8	Garlic < 4 times/wk and psychological index < 33 and cereal < 200 g/wk and milk < 1 time/wk	3.95 (2.79‐5.58)	**0.000**
9	Garlic < 4 times/wk and psychological index ≥ 33	7.97 (5.26‐12.07)	**0.000**

Bold values indicate statistical significance after Bonferroni correction.

Abbreviations: CI, confidence interval; OR, odds ratio; OR_adj_, adjusted by age, educational level, marriage status and family history of cancer.

### 3.6 Association between polymorphisms and clinical characteristics

Next, we calculated the associations of polymorphisms in *CYP27B1* and *IL‐13* with clinical characteristics in BC patients (Table S5‐1 and Table S5‐2). Tumor location, maximum diameter, molecular classification, ER status, PR status, HER2 status, TNM stage, and CA15‐3 were analyzed for this calculation and TNM stage was classified according to the eighth edition of American Joint Committee on Cancer TNM staging system.^21^ We obtained significant correlations between *IL‐13* rs847 and TNM stage (*P* = 0.041). TNM stage was also significantly associated with rs848 (*P* = 0.018) and rs1295685 (*P* = 0.004) in *IL‐13*. No significant differences were discovered with regard to tumor location, maximum diameter, molecular classification, ER status, PR status, HER2 status, or CA15‐3.

## 4 DISCUSSION

In this study, we analyzed multiple exposures of environmental factors and polymorphisms in *CYP27B1* and *IL‐13* with the risk of BC. Noteworthy findings are the significant combined effects between environmental factors (such as garlic intake, physical activity, and psychological index) and polymorphisms in *CYP27B1* (rs10877012 and rs4646536) and *IL‐13* (rs847, rs848, and rs1295685) on BC. Five combining exposure models were suggested to have a significant increased risk of BC.

Several environmental factors were observed to have significant associations with BC risk, including physical activity, psychological index, and the consumption of cereal, garlic, poultry, pork, milk, soybean, and overnight food. This could be explained by a number of reasons. For instance, plant lignans in cereal were previously shown to stimulate the production of enterolactone and butyric acid, which has antitumor activity.^22^ Mathiasen et al^23^ suggested that **c**alcium in milk regulated apoptosis triggered by vitamin D compounds in BC cells, while a cell line study indicated that soybean reduced the growth of BC lines MCF‐7 and MDA‐MB‐231 by silencing JMJD5.^24^ 27‐hydroxycholesterol as the metabolite of cholesterol contained in pork was found to stimulate the xenograft growth of MCF‐7 cells in mice and increase the risk of ER^+^ BC.^25^ Serum cholesterol is also closely related to biosynthesis, which increases the risk of BC when elevated.^26^ Moreover, improper storage of overnight food can lead to the formation of nitrites, which interact with folic acid to increase the risk of BC.^27^

Major organosulfur compounds found in garlic oil, such as diallyl disulfide and diallyl trisulfide, were proposed to suppress BC by halting DNA formation, inhibiting the production of reactive oxygen species, regulating cell cycle arrest, and inducing apoptosis.^28^, ^29^ Garlic was also shown to play a critical role in maintaining homeostasis of the immune system.^30^ Hodge et al^31^ confirmed that fresh garlic extract played a role in suppressing Th1 and inflammatory cytokine production, as well as upregulating the production of IL‐10. Moreover, aged garlic extract and its product N‐α‐(1‐deoxy‐D‐fructos‐1‐yl)‐L‐arginine alleviated neuroinflammatory responses in BV‐2 microglia by regulating gene expression.^32^ Additionally, animal work indicated that protein compositions extracted from fresh garlic bulbs restrained tumor growth by elevating CD8^+^ T cell infiltration into the BC site.^33^ In keeping with these findings, our study indicated that garlic is an independent protective factor of BC, with an intake of ≥4 times/wk significantly decreasing the risk of BC (OR_adj_ = 0.23, 95% CI = 0.15‐0.36). Moreover, garlic also decreased BC risk when combined with all three *IL‐13* SNPs, suggesting that individuals with *IL‐13* variants could benefit from further protection against BC by consuming garlic.

Physical activity has been shown to decrease the production of proinflammatory cytokines in adipose tissue by reducing obesity.^34^ Steensberg et al^35^ reported that increasing exercise contributed to the upregulation of IL‐6, which enhanced the level of antiinflammatory cytokines IL‐1α and IL‐10. Furthermore, IL‐6 inhibited the expression of tumor necrosis factor‐α. Mammographic density is considered a risk element for BC, being associated with tumor size, lymph node status, and lymphatic or vascular invasion,^36^ while participating in physical activity was found to modify the mammographic density by reducing the level of serum estrogens.^37^, ^38^ Multivariate analysis in the present study showed that physical activity ≥1 time/wk was associated with a reduced risk of BC (OR_adj_ = 0.43, 95% CI = 0.32‐0.60), which is in accordance with previous work.^39^, ^40^ Moreover, the results of combined effects for physical activity and SNPs in *CYP27B1* and *IL‐13* showed that genetic variants and taking more exercise collectively decreased susceptibility to BC. Thus, we recommend that carriers of these genetic variants participate in physical exercise to minimize their BC risk.

Analysis of the psychological index and BC risk has hinted at a positive correlation.^41^ Psychological stress increases the level of glucocorticoids, and reduces the ability to repair DNA damage by increasing the production of reactive oxygen species/reactive nitrogen species in BC.^42^, ^43^ Irwin et al reported that psychological changes related to life events reduced the activity of NK cells.^44^ These cells not only mediate the growth and metastasis of BC cells but also play a critical role in antiviral immunity as innate immune effector lymphocytes.^45^, ^46^ Similarly, Levy et al^47^ showed that the levels of NK cells were decreased in BC patients with psychological stress. Our population‐based case‐control study observed that a higher psychological index score (≥33) increased the risk of BC, while significant combined effects were detected between the psychological index and the three *IL‐13* SNPs. Together, these results suggest that maintaining a positive mental state is important for individuals with variant genotypes (AG + GG carriers in rs847, GG + GT carriers in rs848, and CC + CT carriers in rs1295685) to help prevent BC.

Modification of the carcinogenic process occurs both through endogenous biological changes and exogenous exposures, while genetic variation may change an individual's vulnerability to subsequent exposures. Therefore, all exposures should be considered as a whole rather than as individual effects from independent factors. For this reason, we introduced CART and combined it with logistic regression analysis to assess the risk of several possible exposure combination patterns. The classification and regression tree is a recursive method for dividing data sets into two disjointed parts by minimizing the heterogeneity within each outcome.^48^ We analyzed exposure combination models in the risk of BC based on the result of the binary tree with strong generalization ability produced by CART. This provided risk assessments for different dietary and lifestyle combinations. For example, compared with model 1 (consuming garlic ≥ 4 times/wk), individuals with exposure model 6 showed the highest risk of BC. Thus, we recommend that women should avoid exposure patterns associated with high risk.

Research into the link between clinical parameters and the important immunomodulatory cytokine *IL‐13* is limited. Yang et al^49^ reported that Th17 cells, effector CD4(+) T cells involved in inflammation, were related to a low TNM stage so may play an anticancer role in BC. We observed significant associations between TNM stage and the three *IL‐13* polymorphisms (all *P* values < 0.05), suggesting that they could be potential biomarkers of TNM stage in BC.

The present study has a number of limitations which should be taken into account when drawing any conclusions. First, recall bias may have been inevitable when collecting information about environmental factors, although we tried to minimize this. Second, the method of counting food and lifestyle frequencies may have limited the food intake or the ability to perform precise calculations. Third, the sample size may have been insufficient for the analysis of some multiple exposure models. Finally, the results of this retrospective study are limited to Chinese women, so further prospective evidence is needed to validate them in a larger population.

## 5 CONCLUSION

In summary, we report significant combined effects of polymorphisms in *CYP27B1* and *IL‐13* with environmental factors in the risk of BC although these polymorphisms had no significant impact alone. CART analysis suggested important changes that could be made to dietary and behavioral patterns to reduce the risk of BC development.

## CONFLICT OF INTERESTS

The authors have declared that no conflict of interest exists.
