# Supplementary material for: Multiple exposure to environmental factors and variations in CYP27B1 and the microRNA‐binding site of IL‐13 are associated with breast cancer risk
Source: Cancer Med. 2019 Apr 30;8(6):3237–49. doi: 10.1002/cam4.2202 (PMC6558484; doi:10.1002/cam4.2202)
Supplement: Supplementary file 1 [file CAM4-8-3237-s001.docx]

**Table S1. The location of involved SNPs in *CYP27B1* and the predicted biological impact of candidate SNPs located in microRNA-binding site of *IL-13***

| **dbSNP ID** | **Gene** | **Chr** | **Chr Position** | **Location** | **Nucleotide Change** | **Predicted miRNA binding** | **ΔΔG**^b^ | **∣ΔΔG_tot_∣**^c^ |
| --- | --- | --- | --- | --- | --- | --- | --- | --- |
| rs4646536 | *CYP27B1* | 12 | 58157988 | Intron | A>G |  |  |  |
| rs10877012 | *CYP27B1* | 12 | 58162085 | 5' near gene | [G>T](http://www.ncbi.nlm.nih.gov/projects/sviewer/?id=NC_000012.11&search=NC_000012.11:g.58162085G%3ET&v=1:100&content=5) |  |  |  |
| rs847^a^ | *IL-13* | 5 | 131996669 | 3' UTR | [T>C](http://www.ncbi.nlm.nih.gov/projects/sviewer/?id=NC_000005.9&search=NC_000005.9:g.131996669T%3EC&v=1:100&content=5) | mir-98  let-7i  let-7f  let-7g- | 23.5  28.8  29.9  30.7 | 112.9 |
| rs848^a^ | *IL-13* | 5 | 131996500 | 3' UTR | [A>C](http://www.ncbi.nlm.nih.gov/projects/sviewer/?id=NC_000005.9&search=NC_000005.9:g.131996500A%3EC&v=1:100&content=5) | mir-558  mir-595  mir-621  mir-632  let-7i  let-7d  let-7f  let-7g  mir-98 | 27.3  18.3  14.7  21.2  17.1  17.1  12.5  17.1  14.5 | 159.8 |
| rs1295685^a^ | *IL-13* | 5 | 131996445 | 3' UTR | [A>G](http://www.ncbi.nlm.nih.gov/projects/sviewer/?id=NC_000005.9&search=NC_000005.9:g.131996445A%3EG&v=1:100&content=5) | mir-621 | 26.1 | 26.1 |

^a^SNPs located in IL13 with predicted ΔΔG >3 kJ/mol and MAF>5% (MAF, minimum allele frequency); ^b^ΔΔG, DDG, difference of DG between the two alleles (wild-type allele DG – variant allele DG); ^c^|ΔΔG_tot_|, the sum of all the |ΔΔG|s. DG, the Gibbs free energy.

**Table S2.** **Hardy–Weinberg equilibrium test for genotypes distribution in controls**

| **Gene Genotype (SNP)** | **Control No. (%)** | **χ^2^** | ***P-value* _H-W_^a^** |
| --- | --- | --- | --- |
| ***CYP27B1 G>T* (rs10877012 )** | | 2.25 | 0.325 |
| GG | 277 (40.50) |  |  |
| GT | 302 (44.15) |  |  |
| TT | 105 (15.35) |  |  |
| ***CYP27B1 C>T* ( rs4646536)** | | 2.38 | 0.304 |
| CC | 278 (40.47) |  |  |
| CT | 303 (44.10) |  |  |
| TT | 106 (15.43) |  |  |
| ***IL-13 A>G* (rs847)** | | 4.22 | 0.121 |
| AA | 328 (47.61) |  |  |
| AG | 311 (45.14) |  |  |
| GG | 50 (7.26) |  |  |
| ***IL-13 G>T* (rs848)** | | 0.37 | 0.831 |
| GG | 329 (47.85) |  |  |
| GT | 297 (43.56) |  |  |
| TT | 60 (8.59) |  |  |
| ***IL-13 C>T* (rs1295685)** | | 0.41 | 0.814 |
| CC | 324 (47.34) |  |  |
| CT | 298 (43.20) |  |  |
| TT | 61 (9.47) |  |  |

^a^*P*_H-W_: *P* value for Hardy- Weinberg equilibrium test.

**Table S3. Association analyses between the haplotypes of *CYP27B1, IL-13* and the risk of breast cancer**

| **Haplotypes^a^** | **Cases No. (%)** | **Controls No. (%)** | **OR (95% CI)** | ***P* value** | ***P*^*^ value** |
| --- | --- | --- | --- | --- | --- |
| *CYP27B1* (rs4646536, rs10877012) | |  |  |  |  |
| G-C | 888 (65.30) | 850(62.40) | 1.13 (0.96-1.32) | 0.135 | 0.540 |
| T-T | 469 (34.50) | 506(37.20) | 0.89 (0.76-1.04) | 0.135 | 0.540 |
| *IL-13* (rs847, rs848, rs1295685) | |  |  |  |  |
| A-G-C | 881 (67.00) | 907(66.80) | 0.98 (0.83-1.17) | 0.856 | 3.423 |
| G-T-T | 368 (28.00) | 373(27.50) | 1.02 (0.86-1.21) | 0.856 | 3.423 |

Note: Halpotype frequency <0.03 in both control and case has been dropped.

*P**: *P* values after Bonferroni correction.

| **Table S4. Univariate and multivariate analyses for the associations between environment factors and breast cancer risk** | | | | | | | |
| --- | --- | --- | --- | --- | --- | --- | --- |
| **Variables** | **Cases**  **No. (%)** | **Controls**  **No. (%)** | **Univeriate analysis** | |  | **Multivariate analysis** | |
|  |  |  | **OR_adj_** | ***P* value** |  | **OR_adj_** | ***P* value** |
|  |  |  | **(95%CI)** |  |  | **(95%CI)** |  |
| **Cereals (g/week)** |  |  |  |  |  |  |  |
| <50 | 336 (57.05) | 308 (43.44) | 1.00 |  |  | 1.00 |  |
| 50-100 | 158 (26.83) | 186 (26.23) | 0.77 (0.57-1.02) | 0.071 |  | 0.90(0.63-1.28) | 0.552 |
| 100-200 | 59 (10.02) | 104 (14.67) | 0.54 (0.37-0.80) | **0.002** |  | 0.67(0.42-1.06) | 0.087 |
| >200 | 36 (6.11) | 111 (15.66) | 0.36 (0.23-0.55) | **0.000** |  | 0.33(0.20-0.56) | **0.000** |
| **Garlic (times/week)** |  |  |  |  |  |  |  |
| Rarely | 307 (44.43) | 212 (30.03) | 1.00 |  |  | 1.00 |  |
| 1-3 | 303 (43.85) | 268 (37.96) | 0.82 (0.63-1.06) | 0.133 |  | 0.93(0.67-1.28) | 0.649 |
| ≥4 | 81 (11.72) | 226 (32.01) | 0.24 (0.17-0.34) | **0.000** |  | 0.23(0.15-0.36) | **0.000** |
| **Poultry (g/week)** |  |  |  |  |  |  |  |
| 0 | 390 (58.38) | 290 (43.41) | 1.00 |  |  | 1.00 |  |
| <250 | 227 (33.98) | 300 (44.91) | 0.52 (0.41-0.68) | **0.000** |  | 0.44(0.31-0.61) | **0.000** |
| ≥250 | 51 (7.63) | 78 (11.68) | 0.48 (0.31-0.74) | **0.001** |  | 0.38(0.22-0.66) | **0.001** |
| **Pork (g/week)** |  |  |  |  |  |  |  |
| 0 | 93 (13.96) | 128 (18.99) | 1.00 |  |  | 1.00 |  |
| <250 | 307 (46.10) | 288 (42.73) | 1.59 (1.12-2.24) | **0.009** |  | 2.27(1.45-3.58) | **0.000** |
| ≥250 | 266 (39.94) | 258 (38.28) | 1.40 (0.98-1.99) | 0.065 |  | 2.25(1.40-3.60) | **0.001** |
| **Fish (times/month)** |  |  |  |  |  |  |  |
| No | 211 (30.89) | 219 (31.11) | 1.00 |  |  | 1.00 |  |
| ≤1 | 201 (29.43) | 198 (28.13) | 0.82 (0.60-0.12) | **0.208** |  | 0.95(0.64-1.42) | 0.801 |
| >1 | 271 (39.68) | 287 (40.77) | 0.72 (0.54-0.96) | **0.024** |  | 0.95(0.65-1.39) | 0.795 |
| **Milk (times/week)** |  |  |  |  |  |  |  |
| 0 | 434 (63.92) | 343 (50.52) | 1.00 |  |  | 1.00 |  |
| ≤2 | 81 (11.93) | 119 (17.53) | 0.49 (0.35-0.70) | **0.000** |  | 0.50(0.32-0.79) | **0.003** |
| ≥3 | 164 (24.15) | 245 (36.08) | 0.54 (0.42-0.71) | **0.000** |  | 0.54(0.38-0.76) | **0.000** |
| **Soybean (times/week)** |  |  |  |  |  |  |  |
| No | 133 (20.00) | 91 (12.80) | 1.00 |  |  | 1.00 |  |
| ≤1 | 172 (25.86) | 189 (26.58) | 0.61 (0.42-0.89) | **0.010** |  | 0.57(0.35-0.91) | **0.019** |
| 2-3 | 232 (34.89) | 269 (37.83) | 0.56 (0.39-0.79) | **0.001** |  | 0.55(0.35-0.86) | **0.009** |
| ≥4 | 128 (19.25) | 162 (22.78) | 0.55 (0.37-0.81) | **0.002** |  | 0.60(0.37-0.99) | **0.046** |
| **Overnight food^a^ (times/week)** |  |  |  |  |  |  |  |
| No | 117 (16.98) | 174 (24.79) | 1.00 |  |  | 1.00 |  |
| 1-3 | 294 (42.97) | 313 (44.59) | 1.42 (1.04-1.95) | **0.029** |  | 1.07(0.72-1.60) | 0.741 |
| >3 | 278 (40.35) | 215 (30.63) | 2.23 (1.61-3.10) | **0.000** |  | 1.64(1.08-2.51) | **0.021** |
| **Physical activity (times/week)** |  |  |  |  |  |  |  |
| <1 | 477 (69.43) | 305 (49.51) | 1.00 |  |  | 1.00 |  |
| ≥1 | 210 (30.57) | 311 (50.49) | 0.43 (0.34-0.56) | **0.000** |  | 0.43(0.32-0.60) | **0.000** |
| **Psychological index^b^** |  |  |  |  |  |  |  |
| ≤15 | 121 (17.46) | 149 (21.56) | 1.00 |  |  | 1.00 |  |
| 16-33 | 380 (54.83) | 452 (65.41) | 1.07 (0.79-1.50) | 0.654 |  | 0.94(0.64-1.39) | 0.759 |
| ≥33 | 192 (27.71) | 90 (13.02) | 2.59 (1.77-3.79) | **0.000** |  | 2.39(1.48-3.86) | **0.000** |
| **Egg (times/week)** |  |  |  |  |  |  |  |
| No  ≤3 | 85 (12.30)  200 (28.94) | 81 (11.44)  257 (36.30) | 1.00  0.72(0.49-1.06) | 0.099 |  |  |  |
| >3 | 406 (58.76) | 370 (52.26) | 1.07(0.74-1.55) | 0.718 |  |  |  |
| **Canned fruit (times/month)** |  |  |  | 0.210 |  |  |  |
| <1 | 577 (83.74) | 605 (87.94) | 1.00 |  |  |  |  |
| ≥1 | 112 (16.26) | 83 (12.06) | 1.24(0.89-1.72) |  |  |  |  |
| **Canned meat (times/month)** |  |  |  | 0.262 |  |  |  |
| <1 | 628 (91.15) | 648 (94.46) | 1.00 |  |  |  |  |
| ≥1 | 61 (8.85) | 38 (5.54) | 1.31(0.82-2.08) |  |  |  |  |
| **Coffee^c^** |  |  |  | 0.062 |  |  |  |
| Yes | 37 (5.36) | 37 (5.22) | 1.00 |  |  |  |  |
| No | 653 (94.64) | 672 (94.78) | 1.66(0.98-2.84) |  |  |  |  |
| **Carbonated drinks^d^** |  |  |  | 0.228 |  |  |  |
| Yes | 27 (3.91) | 30 (4.24) | 1.00 |  |  |  |  |
| No | 663 (96.09) | 678 (95.76) | 1.45(0.79-2.67) |  |  |  |  |
| **Juice^d^** |  |  |  | 0.570 |  |  |  |
| Yes | 48 (6.96) | 42 (6.12) | 1.00 |  |  |  |  |
| No | 642 (93.04) | 644 (93.88) | 1.15(0.72-1.83) |  |  |  |  |
| **Smoke^e^** |  |  |  | 0.234 |  |  |  |
| Yes | 92 (13.33) | 109 (15.40) | 1.00 |  |  |  |  |
| No | 598 (86.67) | 599 (84.60) | 0.82(0.59-1.14) |  |  |  |  |
| **Alcohol (times/month)** |  |  |  | 0.056 |  |  |  |
| <1 | 602 (88.53) | 583 (90.53) | 1.00 |  |  |  |  |
| ≥1 | 78 (11.47) | 61 (9.47) | 0.68(0.46-1.01) |  |  |  |  |

OR_adj_ : adjusted for age, educational level, marriage status and family history.

^a^Overnight food, the vegetables, eggs, meat that have been cooked and left overnight.

^b^Psychological index was evaluated using psychosocial stress survey for groups (PSSG).

^c^Coffee, drinking at least one cup per month for more than 3 months

^d^Carbonated drinks and ^d^Juice**,** drinking at least one cup per week for more than 3 months

^e^Smoke, smoking at least one per day for more than 6 weeks

**Table S5-1. Association of *CYP27B1* polymorphisms and the clinical characteristics of breast cancer patients**

| **Characteristic** |  | **rs10877012^a^** | | | ***P***  **value** | **rs4646536^a^** | | | ***P***  **value** |
| --- | --- | --- | --- | --- | --- | --- | --- | --- | --- |
|  | **Total (%)** | **GG No.(%)** | **GT No.(%)** | **TT No.(%)** |  | **CC No.(%)** | **CT No.(%)** | **TT No.(%)** |  |
| **Location** |  |  |  |  | 0.906 |  |  |  | 0.848 |
| Left | 328 (52.23) | 143 (43.87) | 139 (42.64) | 44 (13.50) |  | 142(43.56) | 139 (42.64) | 45 (13.80) |  |
| Right | 284 (45.22) | 126 (45.32) | 122 (43.88) | 30 (10.79) |  | 129(46.40) | 119 (42.81) | 30 (10.79) |  |
| Bilateral | 16 (2.55) | 7 (43.75) | 7 (43.75) | 2 (12.50) |  | 7(43.75) | 7 (43.75) | 2 (12.50) |  |
| **Maximum diameter(mm)** |  |  |  |  | 0.264 |  |  |  | 0.319 |
| ≤ 20 | 281 (44.96) | 117 (42.09) | 124 (44.60) | 37 (13.31) |  | 119(42.81) | 122 (43.88) | 37 (13.31) |  |
| 20-50 | 338 (54.08) | 158 (47.45) | 138 (41.44) | 37 (11.11) |  | 158(47.45) | 137 (41.14) | 38 (11.41) |  |
| ≥ 50 | 6 (0.96) | 1 (16.67) | 3 (50.00) | 2 (33.33) |  | 1(16.67) | 3 (50.00) | 2 (33.33) |  |
| **Molecular classification** |  |  |  |  | 0.797 |  |  |  | 0.817 |
| Luminal A | 104 (16.64) | 38 (38.00) | 48 (48.00) | 14 (14.00) |  | 39(39.00) | 46 (46.00) | 15 (15.00) |  |
| Luminal B | 444 (71.04) | 204 (46.26) | 186 (42.18) | 51 (11.56) |  | 205(46.49) | 185 (41.95) | 51 (11.56) |  |
| HER-2(+) | 15 (2.40) | 5 (35.71) | 7 (50.00) | 2 (14.29) |  | 5(35.71) | 7 (50.00) | 2 (14.29) |  |
| Basal-like | 62 (9.92) | 26 (41.94) | 27 (43.55) | 9 (14.52) |  | 26(41.94) | 27 (43.55) | 9 (14.52) |  |
| **ER status** |  |  |  |  | 0.877 |  |  |  | 0.306 |
| Positive | 418 (68.52) | 185 (44.26) | 179 (42.82) | 54 (12.91) |  | 176 (45.71) | 167 (43.38) | 42 (10.91) |  |
| Negative | 192 (31.48) | 87 (45.31) | 83 (43.23) | 22 (11.46) |  | 99 (42.67) | 98 (42.24) | 35 (15.09) |  |
| **PR status** |  |  |  |  | 0.470 |  |  |  | 0.857 |
| Positive | 365 (59.83) | 158 (43.29) | 164 (44.93) | 43 (11.78) |  | 187 (44.63) | 177 (42.24) | 55 (13.13) |  |
| Negative | 245 (40.16) | 114 (46.53) | 98 (40.00) | 33 (13.47) |  | 87 (45.55) | 82 (42.93) | 22 (11.52) |  |
| **HER2 status** |  |  |  |  | 0.369 |  |  |  | 0.442 |
| Positive | 385 (62.40) | 175 (45.45) | 168 (43.64) | 42 (10.91) |  | 159 (43.44) | 163 (44.54) | 44 (12.02) |  |
| Negative | 232 (37.60) | 98 (42.24) | 100 (43.10) | 134 (57.76) |  | 115 (47.13) | 96 (39.34) | 33 (13.52) |  |
| **TNM stage^c^** |  |  |  |  | 0.106 |  |  |  | 0.087 |
| 0-I | 170 (27.07) | 64 (38.46) | 85(50.30) | 20 (11.83) |  | 65(38.46) | 84 (49.70) | 20 (11.83) |  |
| II | 316 (50.32) | 146 (47.25) | 130 (42.07) | 33 (10.68) |  | 147(47.57) | 129 (41.75) | 33 (10.68) |  |
| III-IV | 142 (22.61) | 65 (45.78) | 54 (38.03) | 23 (16.20) |  | 65(45.78) | 53 (45.77) | 24 (16.90) |  |
| **CA15-3(U/ml)** |  |  |  |  | 0.678 |  |  |  | 0.677 |
| ≤100 | 504 (99.41) | 219 (43.89) | 224 (44.89) | 56 (11.22) |  | 219(43.89) | 223 (44.69) | 57 (11.42) |  |
| >100 | 3 (0.59) | 2 (66.67) | 1 (33.33) | 0 (0.00) |  | 2(66.67) | 1 (33.33) | 0 (0.00) |  |

^a^Missing data, 8 for rs10877012 and 8 DNA samples were not successful for rs4646536. ^b^Bold means statistical significance.

^c^TNM stage was classified according to the 8^th^ edition of American Joint Committee on Cancer. TNM staging system, 0-I indicates stage 0, stage IA and stage IB; II indicates stage IIA and stage IIB; III-IV indicates stage IIIA, stage IIIB, stage IIIC and stage IV.

**Table S5-2. Association of *IL-13* polymorphisms and the clinical characteristics of breast cancer patients**

| **Characteristic** |  | **rs847^a^** | | | ***P***  **value** | **rs848^a^** | | | ***P value*** | **rs1295685^a^** | | | ***P***  **value** |
| --- | --- | --- | --- | --- | --- | --- | --- | --- | --- | --- | --- | --- | --- |
|  | **Total (%)** | **AA No.(%)** | **AG No.(%)** | **GG No.(%)** |  | **GG No.(%)** | **GT No.(%)** | **TT No.(%)** |  | **CC No.(%)** | **CT No.(%)** | **TT No.(%)** |  |
| **Location** |  |  |  |  | 0.756 |  |  |  | 0.613 |  |  |  | 0.620 |
| Left | 328 (52.23) | 154 (48.28) | 135 (42.32) | 30 (9.40) |  | 155 (47.99) | 140 (43.34) | 28 (8.67) |  | 156 (48.15) | 139 (42.90) | 29 (8.95) |  |
| right | 284 (45.22) | 125 (46.13) | 120 (44.28) | 26 (9.59) |  | 127 (45.68) | 122 (43.88) | 29 (10.43) |  | 123 (44.24) | 123 (44.24) | 32 (11.51) |  |
| Bilateral | 16 (2.55) | 8 (57.14) | 6 (42.86) | 0 (0.00) |  | 9 (60.00) | 6 (40.00) | 0 (0.00) |  | 9 (60.00) | 5 (33.33) | 1 (6.67) |  |
| **Maximum diameter(mm)** | |  |  |  | 0.757 |  |  |  | 0.705 |  |  |  | 0.338 |
| ≤20 | 281 (44.96) | 125 (46.64) | 118 (44.03) | 25 (9.33) |  | 126 (46.49) | 118 (43.54) | 27 (9.96) |  | 128 (47.06) | 114 (41.91) | 30 (11.03) |  |
| 20-50 | 338 (54.08) | 156 (47.71) | 141 (43.12) | 30 (9.17) |  | 159 (47.32) | 148 (44.05) | 29 (8.63) |  | 156 (46.43) | 150 (44.64) | 30 (8.93) |  |
| ≥50 | 6 (0.96) | 4 (66.67) | 1 (16.67) | 1 (16.67) |  | 4 (66.67) | 1 (16.67) | 1 (16.67) |  | 2 (33.33) | 2 (33.33) | 2 (33.33) |  |
| **Molecular classification** | |  |  |  | 0.642 |  |  |  | 0.838 |  |  |  | 0.855 |
| LuminalA | 104 (16.64) | 47 (47.47) | 40 (40.40) | 12 (12.12) |  | 47 (47.00) | 42 (42.00) | 11 (11.00) |  | 45 (44.55) | 44 (43.56) | 12 (11.88) |  |
| LuminalB | 444 (71.04) | 201 (47.18) | 185 (43.43) | 40 (9.39) |  | 205 (47.02) | 190 (43.58) | 41 (9.40) |  | 205 (46.91) | 188 (43.02) | 44 (10.07) |  |
| HER-2(+) | 15 (2.40) | 7 (46.67) | 8 (53.33) | 0 (0.00) |  | 7 (46.67) | 8 (53.33) | 0 (0.00) |  | 7 (46.67) | 8 (53.33) | 0 (0.00) |  |
| Basal-like | 62 (9.92) | 31 (50.82) | 27 (44.26) | 3 (4.92) |  | 31 (50.00) | 27 (43.55) | 4 (6.45) |  | 30 (49.18) | 26 (42.62) | 5 (8.20) |  |
| **ER status** |  |  |  |  | 0.587 |  |  |  | 0.732 |  |  |  | 0.735 |
| Positive | 406  (68.35) | 194  (47.78) | 172  (42.63) | 40  (9.85) |  | 198  (47.60) | 178  (42.78) | 40  (9.62) |  | 198  (47.37) | 177  (42.34) | 43  (10.29) |  |
| Negative | 188  (31.65) | 89  (47.34) | 85  (45.21) | 14  (7.45) |  | 89  (46.84) | 86  (45.26) | 15  (7.89) |  | 86  (45.50) | 86  (45.50) | 17  (8.99) |  |
| **PR status** |  |  |  |  | 0.111 |  |  |  | 0.144 |  |  |  | 0.219 |
| Positive | 353  (59.43) | 168  (47.59) | 146  (41.36) | 39  (11.05) |  | 174  (47.93) | 150  (41.32) | 39  (10.74) |  | 171  (46.85) | 152  (41.64) | 42  (11.51) |  |
| Negative | 241  (40.57) | 115  (47.72) | 111  (46.06) | 15  (6.22) |  | 113  (46.50) | 114  (46.91) | 16  (6.58) |  | 113  (46.69) | 111  (45.87) | 18  (7.44) |  |
| **HER2 status** |  |  |  |  | 0.910 |  |  |  | 0.957 |  |  |  | 0.671 |
| Positive | 375  (62.40) | 178  (47.47) | 164  (43.33) | 33  (8.8) |  | 182  (47.64) | 166  (43.46) | 34  (8.90) |  | 184  (48.04) | 163  (42.56) | 36  (9.40) |  |
| Negative | 226  (37.60) | 108  (47.79) | 96  (42.48) | 22  (9.73) |  | 108  (46.75) | 101  (43.72) | 22  (9.52) |  | 103  (44.59) | 103  (44.59) | 25  (10.82) |  |
| **TNM stage^c^** |  |  |  |  | **0.041^b^** |  |  |  | **0.018^b^** |  |  |  | **0.004^b^** |
| 0-Ⅰ | 170 (27.07) | 70 (43.48) | 75 (46.58) | 16 (9.94) |  | 71 (43.29) | 77 (46.95) | 16 (9.76) |  | 72 (43.64) | 75 (45.45) | 18 (10.91) |  |
| Ⅱ | 316 (50.32) | 152 (50.33) | 131 (43.38) | 19 (6.29) |  | 155 (49.84) | 137 (44.05) | 19 (6.11) |  | 154 (49.52) | 138 (44.37) | 19 (6.11) |  |
| Ⅲ | 123 (19.59) | 59 (48.36) | 44 (36.07) | 19 (15.57) |  | 59 (48.36) | 45 (36.89) | 18 (14.75) |  | 57 (46.72) | 44 (36.07) | 21 (17.21) |  |
| Ⅳ | 19 (3.03) | 6 (31.58) | 11 (57.89) | 2 (10.53) |  | 6 (31.58) | 9 (47.37) | 4 (21.05) |  | 5 (26.32) | 10 (52.63) | 4 (21.05) |  |
| **CA15-3(U/ml)** |  |  |  |  | 0.764 |  |  |  | 0.763 |  |  |  | 0.752 |
| ≤100 | 504 (99.41) | 234 (48.35) | 206 (42.56) | 44 (9.09) |  | 238 (48.18) | 212 (42.91) | 44 (8.91) |  | 237 (47.98) | 208 (42.11) | 49 (9.92) |  |
| >100 | 3 (0.59) | 2 (66.67) | 1 (33.33) | 0 (0.00) |  | 2 (66.67) | 1 (33.33) | 0 (0.00) |  | 2 (66.67) | 1 (33.33) | 0 (0.00) |  |

^a^Missing data, 24 for rs847; 12 for rs848; 11 for rs1295685. ^b^Bold means statistical significance.

^c^TNM stage was classified according to the 8^th^ edition of American Joint Committee on Cancer. TNM staging system, 0-I indicates stage 0, stage IA and stage IB; II indicates stage IIA and stage IIB; III-IV indicates stage IIIA, stage IIIB, stage IIIC and stage IV.
